# How and When COVID‐19‐related News Consumption Relates to Mental Health Complaints—A Cross‐Sectional Study

**DOI:** 10.1002/hsr2.72999

**Published:** 2026-08-11

**Authors:** H. M. Saidur Rahaman, Rozi Samad

**Affiliations:** ^1^ Department of Psychology Jagannath University Dhaka Bangladesh

**Keywords:** COVID‐19‐related knowledge, COVID‐19‐related news consumption, COVID‐19‐triggered mortality salience, mental health complaints

## Abstract

**Background and Aims:**

Since the emergence of COVID‐19, a substantial body of research has been published regarding its psychological impacts, particularly on young adults. COVID‐19‐related news consumption has been shown to be an important factor in this regard. However, extant research is largely exploratory in nature and lacks a more nuanced examination of this relationship. Against this backdrop, in the present study, we draw on terror management theory to develop and test a moderated mediation model to examine how and when COVID‐19‐related news consumption may relate to young adults' mental health complaints. Specifically, we hypothesize that COVID‐19‐triggered mortality salience moderates the positive indirect association between COVID‐19‐related news consumption and mental health complaints through COVID‐19‐related knowledge, such that the indirect relationship is more pronounced when the level of COVID‐19‐triggered mortality salience is relatively high compared to low.

**Methods:**

A cross‐sectional survey was conducted with 250 student participants from two public universities in the capital of Bangladesh.

**Results:**

Results support our proposed hypotheses. Moderated mediation analysis showed that the hypothesized model accounted for approximately 31% of the variance in mental health complaints.

**Conclusion:**

Our study elucidates a nuanced mechanism of how and when COVID‐19‐related news consumption relates to mental health complaints by presenting a moderated mediation model. Our study findings may inform further research related to the global pandemic and mental health.

## 1 Introduction

The world has seen the dire consequences of COVID‐19 for people in all spheres of life: personal, social, political, and economic [1, 2, 3, 4, 5]. In particular, COVID‐19 has largely impacted the young population by increasing their stress and diminishing their coping [6, 7], depression, and anxiety [8, 9], and smartphone addiction [10]. Specifically, studies have shown the negative consequences of COVID‐19‐related news consumption on psychological distress [11, 12], parental mental health [13], and depression and PTSD for individuals who experienced relatively more severe childhood trauma [14], and depressive and anxiety symptoms [15]. Importantly, COVID‐19‐related news consumption has been linked to the deterioration of young adults' mental health [16]. Specifically, university graduates usually sought information about the virus [17].

However, the extant research does not offer a clear answer to the question of how and under what conditions COVID‐19‐related news consumption may relate to young adults' (i.e., university students) mental health. University students are three times more likely than the general population to experience mental health concerns [18]. As a result, they are viewed as a vulnerable population [19]. Hence, a further understanding of the nuanced nature of the relationship between COVID‐19‐related news consumption and young adults' mental health is needed to develop proper policy implications for tackling future pandemics or disastrous events and maintaining their mental health and well‐being [1].

**Figure 1:**
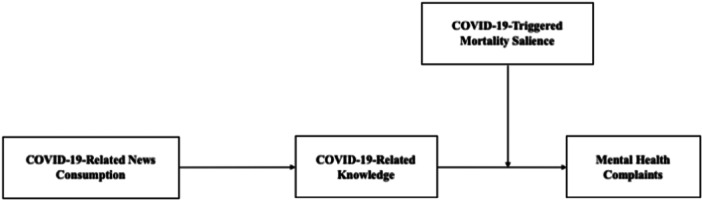
The proposed research model.

Given the above, in the present study, we draw on terror management theory (TMT) [20, 21] and go above and beyond the previous studies to examine how and when COVID‐19‐related news consumption may relate to young adults' (i.e., university students) mental health complaints. As such, we theorize that COVID‐19‐related news consumption operates as a cue of vulnerability and death [2] to young adults, which eventually relates to their mental health complaints via their COVID‐19‐related knowledge. TMT theory further admits that “people differ in their capacity to sustain terror‐assuaging meaning and self‐esteem, and they also derive meaning and self‐esteem from different types of ideas, activities, and experiences” [22], p. 909. Here, we propose COVID‐19‐triggered mortality salience as an individual difference‐based boundary condition for the aforementioned mediating relationship. Specifically, we hypothesize that the mediated relationship between COVID‐19‐related news consumption and mental health complaints, via COVID‐19‐related knowledge, would be stronger for individuals who experience a relatively high level of COVID‐19‐triggered mortality salience compared to their counterparts who experience a relatively low level of COVID‐19‐triggered mortality salience. In all, we develop a moderated mediation‐based theoretical model (see Figure 1).

Our study has two important implications for the relevant research. First, we go above and beyond the extant research (e.g. [11, 12, 13]) to show how and when COVID‐19‐related news consumption may relate to young adults' mental health complaints and contribute to this direction of research. In doing so, we also address the call of Holmes et al. [1], who urge us to do research to better understand the association between news consumption and the mental health of various age groups (i.e., young adults). Furthermore, introducing a mediator and moderator based on the tenets of TMT can be considered a significant theoretical contribution [23]. Second, we contribute to the growing body of research focusing on TMT in the context of COVID‐19 (e.g. [24, 25, 26]). We specifically respond to the call of Pyszczynski et al. [21] to conduct further research (i.e., although some research has been conducted in East Asian countries) in the Asian context. To our knowledge, we are the first to test the TMT theory in a South Asian context (i.e., Bangladesh), focusing on the COVID‐19 situation and contributing to the extension of the theory to a relatively less investigated population. Moreover, scholars also suggest doing further research on mental health in the context of COVID‐19 in other relatively less investigated countries [27].

## 2 Theory and Hypothesis Development

According to TMT [20], people adhere to meaningful worldviews that provide them with a sense of enduring self‐worth because they feel threatened by their own mortality. Hence, TMT provides a suitable theoretical framework for comprehending possible fatal occurrences in life [28]. The theory states that the fear of death causes crippling anxiety [20]. TMT‐based research suggests that being reminded of one's death can cause anxiety and negatively impact one's well‐being [20, 29].

In the context of COVID‐19, people encounter “a constant barrage of threatening information [COVID‐19‐related news consumption]” about the virus, which can operate as a constant reminder of death and peril [2], p. 176). Research suggests that functional impairment of the anxiety buffer, as well as death anxiety, are transdiagnostic susceptibility factors for psychiatric disorders [30, 31]. As per TMT, fear of death and maladaptive coping mechanisms are frequent outcomes when people are unable to adequately manage their existential terror, particularly during the time of COVID‐19, via creating a meaningful and purposeful existence [2]. Mental health is probably impacted by the general public's exposure to and consumption of the news media, which presents contradictory information on the risks that the virus and the pandemic represent. Individuals' views of potential benefits and hazards are shaped by the news media's social influence [12, 32]. Indeed, prior studies have documented that greater news consumption about crisis situations is positively associated with people's mental health [33, 34]. Specifically, news media consumption may become distressing, especially when news media about the event (e.g., new infection, death, limited medical facilities) are repeatedly viewed and difficult to overlook [12]. This repetition may facilitate young adults' COVID‐19‐related knowledge, defined as the “processing of fact or information that allows an accurate awareness of a topic [COVID‐19‐related news]” by virtue of the process of information acquisition [35], p.1384). Such acquisition of knowledge (i.e., this refers to their perceived knowledge) may trigger an individual's mental health vulnerability as they are likely to experience wariness and fear [36]. In all, COVID‐19‐related news consumption may positively associate with COVID‐19‐related knowledge. And, this knowledge may eventually positively relate to mental health complaints. Thus, following the above arguments, we hypothesize the following:

Hypothesis 1 COVID‐19‐related knowledge mediates the relationship between COVID‐19‐related news consumption and mental health complaints, such that COVID‐19‐related news consumption is positively related to COVID‐19‐related knowledge, and COVID‐19‐related knowledge is positively associated with mental health complaints.

TMT theory further suggests that the ability of individuals to maintain terror‐inducing meaning and self‐worth varies, as do the kinds of concepts, pursuits, and experiences that provide them with these qualities [22]. Research shows the differential impacts of mortality salience on individuals' prosocial behavior [37]. Hence, individuals' perceptions of mortality salience (i.e., reminder of death [2]) concerning COVID‐19 can be important in this regard, as it can affect individuals' anxiety about their death and cause enormous anxiety. Furthermore, individual differences exist for COVID‐19‐triggered mortality salience [25]. Taken together, we expect that individuals who have a relatively high level of COVID‐19‐triggered mortality salience may be more susceptible to mental health complaints. Specifically, for them, the positive association between COVID‐19‐related knowledge and mental health complaints can be more pronounced as they may get overwhelmed by the amount of anxiety and become more vulnerable to mental health issues [2]. On the contrary, individuals who experience a relatively low level of COVID‐19‐triggered mortality salience may be less susceptible to mental health issues as they are less likely to be defensive and anxious. Indeed, prior research demonstrates the moderating role of mortality salience (e.g. [24, 38]). Thus, we propose the following hypothesis:

Hypothesis 2 COVID‐19‐triggered mortality salience moderates the positive relationship between COVID‐19‐related knowledge and mental health complaints, such that the relationship is stronger (weaker) when COVID‐19‐triggered mortality salience is perceived to be high (low).

Since the relationship between COVID‐19‐related knowledge and mental health complaints is moderated by COVID‐19‐triggered mortality salience, we also anticipate that COVID‐19‐triggered mortality salience will have an impact on the strength of the indirect relationship between COVID‐19‐related news consumption and mental health complaints through COVID‐19‐related knowledge. Diagrammatically, the relationship looks as follows: COVID‐19‐related news consumption →COVID‐19‐related knowledge X COVID‐19‐triggered mortality salience → Mental health complaints. Thus, we formulate the subsequent hypothesis:

Hypothesis 3 COVID‐19‐triggered mortality salience moderates the indirect relationship between COVID‐19‐related news consumption and mental health complaints such that the indirect relationship will be stronger (weaker) when COVID‐19‐triggered mortality salience is perceived to be high (low).

## 3 Methods

### 3.1 Participant

The study sample consisted of 250 university students (Male=125 and Female=125) from two public universities in the capital of Bangladesh. They averaged 23.70 years (SD = 2.27) of age. Among the participants, 81.2 percent were undergraduate students, 18.8 percent were graduate students, and 2 percent were missing reports. We used a convenience sampling technique and a cross‐sectional design to collect the data. We collected data during the period of October 2021, which was the third wave of the COVID‐19 pandemic in Bangladesh. In collecting the data, we did not apply any exclusion criteria for participants' recruitment.

### 3.2 Measures

We used the following standardized questionnaires in our study. We followed the standard translation‐back‐translation procedure [39] with two subject‐matter experts (i.e., one language expert and one psychology expert) who reviewed the questionnaire items to ensure construct equivalence. The disagreements between them were resolved through further discussion and editing of the translated items with the involvement of one of the authors. Eventually, the finalized and translated questionnaires were administered for the survey (see [25], for a similar approach).

### 3.3 Mental Health Complaints (MHI‐5)

A mental health inventory developed by Berwick et al. [40] was administered to assess mental health complaints. It has five items anchored with a 6‐point Likert scale: *1=never to 6=always*. A sample item was “How much of the time, during the last month, have you been a very nervous person?” (α = 0.85). Following the original scale, items 2 and 4 were reverse‐scored, and a high score indicated more mental health complaints.

### 3.4 COVID‐19‐Triggered Mortality Salience

We measured COVID‐19‐triggered mortality salience by using the items used by Hu et al. [25]. They adopted the items from Grant, Franklin, and Langford's scale [41]. The scale consists of 2 items and is anchored with a 7‐point Likert‐type scale ranging from *1=strongly disagree to 7=strongly agree*. A sample item was “Today, I frequently examined my feelings about COVID‐19 related deaths” (α = 0.85).

### 3.5 COVID‐19‐Related Knowledge

We measured COVID‐19‐related knowledge with a single item used in recent research [35]. The item was anchored on a 5‐point scale: *1=Not at all knowledgeable, 2=slightly knowledgeable, 3=somewhat knowledgeable, 4=moderately knowledgeable, 5=extremely knowledgeable*. The item was “How knowledgeable would you rate yourself with regards to COVID‐19 (e.g., symptoms, how to prevent getting the virus, and prevalence in your state)?”.

### 3.6 COVID‐19‐Related News Consumption

We used a 5‐point (*1=never to 5* = *a great deal*) single‐item scale to measure COVID‐19‐related news consumption [35]. The item was “Since the start of the COVID‐19 pandemic in Bangladesh, how frequently have you followed the news related to the pandemic?”.

### 3.7 Procedure

We followed the standard data collection procedure in the present study. Participants were recruited through classroom recruitment, and they completed a paper‐based survey. At the beginning, we briefed the participants about the general purpose of the study. Then, we further informed them that the investigation was purely academic in nature and their responses to the questionnaires would be kept anonymous and confidential. Lastly, we administered the surveys to the participants who gave their consent. Participants first provided information related to their age, gender, and educational qualification. Then, they rated the questionnaires of the present study. On average, participants needed 15 min to complete the survey. Finally, we thanked them for their participation, and we also provided them with some snacks as a reward. The response rate of the survey was 89 percent.

### 3.8 Analytical Approach

Before testing our hypotheses, we first ran a Harman one‐factor test following recent research (e.g. [42]) and found that a single factor can explain 44.26 percent of the variance, which is less than 50 percent. Hence, we deem that common method variance is not a major concern for our study [43]. To test our whole model, we employed statistical software IBM SPSS 24.0 and macro program PROCESS 2.16.3 developed by Hayes [44] and the bootstrap‐based analytical technique of Edwards and Lambert [45]. Specifically, we used PROCESS model 4 for testing the mediation effect, model 1 for the moderation effect, and model 14 for the overall moderated mediation effect. We employed a bias‐corrected bootstrapping technique in order to get adequate statistical power and robust findings and avoid the issue of not satisfying the assumptions of normality [44, 46]. We did not use any covariates (e.g., age, gender) in running our regression analysis with PROCESS. First, as shown in Figure 1, we examined hypotheses 1 and 2, which are summarized in the overall theoretical model. A model with this structure is known as a second‐stage moderated mediation model [45, 47]. It might not be enough to examine hypotheses 1‐2 by evaluating the importance of individual paths in our model for developing a moderated mediation effect [45, 48]. Therefore, we examined hypothesis 3, which describes the total moderated mediation effect, after obtaining support for our hypotheses 1 and 2. Using 5000 bootstrapped resamples, we assessed the moderating and conditional indirect effect using 95% confidence intervals at one standard deviation above and below the mean. All tests were conducted as two‐tailed, and statistical significance was determined at *p‐values* lower than 0.05.

## 4 Ethical Consideration

For this research, participants provided their informed consent. The researchers state that all methods used in studies with human participants followed national and institutional ethical guidelines, as well as the principles outlined in the Helsinki Declaration. The researchers are committed to maintaining research ethics. The Psychology Department at Jagannath University granted ethical approval, with registration number HREC/JnU/Psy/05 2024 retrospectively, due to the unavailability of such service in a full‐fledged manner during the study execution period. Furthermore, obtaining ethical approval was also not an institutional requirement at that time.

## 5 Results

**Figure 2:**
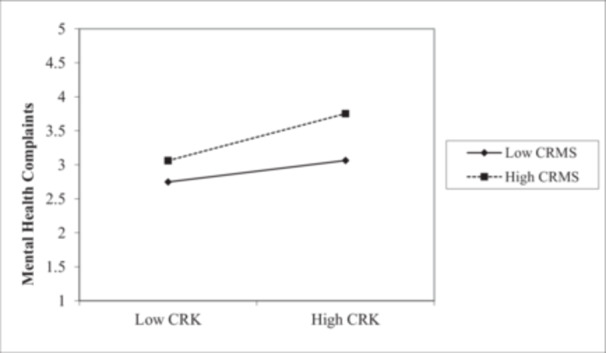
The interaction between COVID‐19‐triggered mortality salience and COVID‐19‐related knowledge on mental health complaints. CRMS = COVID‐19‐triggered mortality salience, CRK = COVID‐19‐related knowledge.

The mean, standard deviation, and correlation values of the study variables are presented in Table 1. We employed a series of regression analyses and found a positive association between COVID‐19‐related news consumption and COVID‐19‐related knowledge (*b* = 0.89, *SE* = 0.05, *p* < 0.01) while controlling for COVID‐19‐related news consumption a positively associated relationship between COVID‐19‐related knowledge and mental health complaints was found (*b* = 0.32, *SE* = 0.10, *p* < 0.05). The coefficient for the indirect effect of COVID‐19‐related news consumption on mental health complaints via COVID‐19‐related knowledge was 0.17 (Boot *SE* = 0.07), and the bias‐corrected 95% confidence interval did not contain 0 (95% *CI* = 0.05 to 0.32), which supports hypothesis 1 (see Table 2). Concerning hypothesis 2 (see Table 3), results show that the interaction term COVID‐19‐related knowledge by COVID‐19‐triggered mortality salience significantly predicts mental health complaints (*b* = 0.09, *SE* = 0.04, *p* < 0.05). For the purpose of investigating the interaction pattern and the moderator's boundary conditions, we further plotted our data and performed a simple slope analysis (see Figure 2). Simple slopes demonstrated that when COVID‐19‐triggered mortality salience was high, COVID‐19‐related knowledge was significantly related to mental health complaints (*b* = 0.41, *SE* = 0.10, *p*< 0.01, 95% *CI* = 0.22 to 0.60). However, when COVID‐19‐triggered mortality salience was low, COVID‐19‐related knowledge was not significantly related to mental health complaints (*b* = 0.09, *SE* = 0.08, 95% *CI* = ‐.07 to 0.25). Thus, hypothesis 2 was supported. For hypothesis 3 (see Table 4), the results indicated that the indirect effect of COVID‐19‐related news consumption on mental health complaints was significant at higher levels of COVID‐19‐triggered mortality salience (conditional indirect effect = 0.28, Boot *SE* = 0.10, 95% *CI* = 0.09 to 0.48). Contrary, at lower levels of COVID‐19‐triggered mortality salience the indirect effect was nonsignificant (conditional indirect effect= 0.006, Boot *SE* = 0.09, 95% *CI* = ‐ 0.18 to 0.19). The index of moderated mediation was also significant (index of moderated mediation = 0.08, Boot SE = 0.04, 95% CI = 0.006 to 0.16). Hence, hypothesis 3 was also supported.

**Table 1:** Descriptive statistics, intercorrelations, and cronbach's alphas of the study variables.

Variables	*M*	SD	1	2	3	4
1. Covid‐19‐related news consumption	3.52	1.05	**—**			
2. Covid‐19‐related knowledge	3.20	1.19	0.78*	**—**		
3. Mental health complaints	3.25	1.17	0.44*	0.42*	**0.85**	
4. Covid‐19‐triggered mortality salience	3.70	1.68	0.57*	0.51*	0.49*	**—**

*Note: N* = 250. Values on the diagonal (bold) are Cronbach's alphas.

* *p* < 0.01.

**Table 2:** Summary of analyses for hypothesis 1.

	Covid‐19‐related knowledge			Mental health complaints		
	*b*	*SE*	*t*	*b*	*SE*	*t*
Covid‐19‐related news consumption	0.89*	0.05	19.49*	0.20**	0.09	2.22**
Covid‐19‐related knowledge				0.32*	0.10	3.18*

	*Indirect Effects of Covid‐19‐Related News Consumption on Mental Health Complaints (through Covid‐19‐Related Knowledge)*			
	*Indirect Effect*	*Boot SE*	*Lower 95% bootstrap confidence interval*	*Higher 95% bootstrap confidence interval*
	0.17	0.07	0.05	0.32

*Note:* Values are unstandardized regression coefficients.

* *p* < 0.01

** **p < 0.05.

**Table 3:** Summary of regression analyses for hypothesis 2.

	Mental health complaint		
	*b*	*SE*	*t*
Covid‐19‐related knowledge	0.25*	0.06	4.06*
Covid‐19‐triggered mortality salience	0.25*	0.04	5.75*
Covid‐19‐related knowledge x Covid‐19‐triggered mortality salience	0.09**	0.04	2.42**
*R* ^*2*^			0.30

	*Conditional Effects of Covid‐19‐Related Knowledge on Mental Health Complaints at Covid‐19‐Triggered Mortality Salience Low versus Moderate versus High*		
	*Effect*	*SE*	*t*
Low (‐1 SD)	0.09	0.08	1.15
Moderate (Mean)	0.25*	0.06	4.06*
High (+ 1 SD)	0.41*	0.10	4.20*

*Note:* Values are unstandardized regression coefficients. Independent variables were mean centered.

* *p* < 0.01

** *p* < 0.05.

**Table 4:** Results for the conditional indirect effect of covid‐related news consumption on mental health complaints (through Covid‐19‐Related Knowledge) at Different Levels of Covid‐19‐Triggered Mortality Salience.

	Covid‐19‐related knowledge		Mental health complaints	
	*b*	*SE*	*b*	*SE*
Covid‐19‐related news consumption	0.89*	0.05	0.15	0.10
Covid‐19‐related knowledge			0.16	0.08
Covid‐19‐triggered mortality salience			0.23*	0.05
Covid‐19‐related knowledge x Covid‐19‐triggered mortality salience			0.09**	0.04
*R^2^*	0.61		0.31	

	*Conditional Indirect Effects at Covid‐19‐Triggered Mortality Salience Low versus Moderate versus High*			
Covid‐19‐triggered mortality salience	*Indirect Effect*	*Boot SE*	*Lower 95% bootstrap confidence interval*	*Higher 95% bootstrap confidence interval*
Low (‐ 1 SD)	0.006	0.09	−0.18	0.19
Moderate (Mean)	0.14	0.07	0.01	0.29
High (+ 1 SD)	0.28	0.10	0.09	0.48

	*Index of Moderated Mediation*			
	*Index*	*Boot SE*	*Lower 95% bootstrap confidence interval*	*Higher 95% bootstrap confidence interval*
Covid‐19‐related knowledge	0.08	0.04	0.006	0.16

*Note:* Values are unstandardized regression coefficients. Independent variables were mean centered.

* *p* < 0.01

** *p* < 0.05.

## 6 Discussion

In the present study, we draw on terror management theory to understand how (i.e., through an increase in COVID‐19‐related knowledge) and when (i.e., depending on the relative level of COVID‐19‐triggered mortality salience) COVID‐19‐related news consumption relates to young adults' mental health complaints. We found that a relatively high level of COVID‐19‐triggered mortality salience strengthens the indirect relationship between COVID‐19‐related news consumption and mental health complaints via COVID‐19‐related knowledge. Below, we are discussing theoretical implications, practical implications, limitations, strengths, and future research directions for our findings.

### 6.1 Theoretical Implications

First, our study findings extend the extant research (e.g. [11, 12, 13]) on news consumption and mental health. Here, we contribute by providing a more nuanced mechanism of the link between COVID‐19‐related news consumption and young adults' mental health complaints. Second, we extend the application of TMT in the context of COVID‐19 to a unique population based in a South Asian country. The extant research mainly examines TMT research in the context of East Asian countries only [21]. In our research, we also demonstrate COVID‐19‐triggered mortality salience as an individual difference variable. That is, not all young adults are equally vulnerable due to their different levels of COVID‐19‐triggered mortality salience. Indeed, research suggests that individuals may be non‐defensive in the context of mortality salience [38]. In our research, we introduce COVID‐19‐triggered mortality salience as a boundary condition, which may provide a plausible explanation for the ambiguous role of COVID‐19‐related knowledge as a mediator for depressive symptoms (see [35]) in extant research and make a meaningful contribution accordingly.

### 6.2 Practical Implications

Our study findings provide important practical implications for managing young adults' mental health during the pandemic and for future pandemics, which are an inevitable part of human history [15]. First, policymakers may take specific steps (i.e., organizing online events, competitions, and events) to occupy young adults with developmental and growth‐oriented activities so that they can spend less time focusing on the media and news [49]. They may also want to utilize public health initiatives and/or clinical interventions [11] to reduce news consumption to evade its dire consequences for mental health. However, we do acknowledge the importance of informed citizenship in this regard. Second, as we show that COVID‐19‐triggered mortality salience works as an individual difference factor, policymakers may also want to organize specific discussions, talks, workshops, and events focusing on minimizing the effect of mortality salience to ensure the well‐being of young adults. Secondarily, during future pandemics, a communication policy may be devised in a way that would ensure the dissemination of reliable, consistent, accurate, and expert‐led real‐time updates through both traditional media and social media to minimize the occurrence of fear due to false and inaccurate information [15], which may help minimize the adversity of mortality salience. Third, our findings also provide important takeaways for university administration. In the time of the pandemic, relevant bodies of the university may also keep in mind the dire consequences of the pandemic on young students' mental health. Specifically, they may become less likely to seek mental health support [50]. Hence, university counseling centers and relevant departments should proactively organize workshops and provide constraint‐free access to mental health services to their students, considering the insights of the current study findings. Research based on Chinese university students shows that they were at high risk of PTSSs and unmet mental health services during the pandemic [50], which further strengthens our arguments for the above. Moreover, research based on the British Columbia province of Canada suggests the increment of mental health service utilization efforts during the pandemic for specific subpopulations such as youth and adolescents [51], which implies the need for more resource allocation for mental health services in Bangladeshi universities for future pandemics. Lastly, for individuals with high mortality salience due to the pandemic, building their self‐esteem, making life experiences meaningful, practicing spirituality, and fostering relationships with family members may help them mitigate the adverse effects of news consumption (see [52]).

### 6.3 Limitations, Strengths, and Future Research Directions

Several key limitations of our research should be acknowledged. First, we collected cross‐sectional data, which may undermine the causal nature of our proposed theoretical model. Common method bias can also be another concern. However, we performed Harman's one‐factor test, and the results show that common method bias can be less likely to be a concern for our dataset [43], but we also acknowledge its limitations. We further swap the predictor (i.e., COVID‐19‐related news consumption) with the outcome variable (i.e., Mental health complaints), while keeping the mediator and the moderator constant in our model, to check whether the results are significant. Given the non‐significant results, we deemed the reverse causality issue less likely in our dataset. Indeed, future research should be conducted by employing experimental and longitudinal designs to establish the causal nature of our theoretical model. Second, we collected data from two public universities in Dhaka city, which may raise concerns regarding the generalization of our findings. Moreover, the sample size was not large enough to represent the whole young population of Bangladesh, and we did not conduct a prior power analysis to determine the sample size. Furthermore, we acknowledge that we did not assess the key regression assumptions. Since we employed a bootstrapping‐based confidence interval (CI), which places fewer constraints on the sample size and does not impose the assumption of normality [46, 48], we deem this issue may not be a serious concern in interpreting the results of our study. To allow for broader generalization, we do, however, advocate for more research (i.e., replicating with a non‐South Asian or post‐pandemic setting) to confirm or extend the results of our study. Third, for two of our study variables, we used single‐item measures, which may raise concerns regarding psychometric properties. However, the specific measures of our study are clearly defined and unidimensional in nature (see [53]), which is deemed permissible in the context of our study. Indeed, future research needs to be executed with more rigorous and robust measures to further validate our theoretical model. Lastly, social desirability bias can also be a potential concern in interpreting our study findings.

The findings of our study offer some interesting avenues for future research. First, recent research suggests that household registration (urban vs. rural) of the Chinese students has an impact on their experience of depression [54], which we could not address in our study. Hence, future research may be conducted exploring the potential influence of demographic factors on mental health. Second, while in our research we only examine the risky pathways linking COVID‐19‐related news consumption to mental health complaints via COVID‐19‐related knowledge, future research can be examined to unravel protective mechanisms, considering our model, as recent research suggests the shielding properties of psychological resilience dealing with stress during the COVID‐19 pandemic [55]. Third, our research provides a nuanced understanding of how and when news consumption can be related to mental health complaints, but cannot specify specific types of media sources. Hence, in concurrence with the initial research findings that different media combinations have a differential impact on people's depression and anxiety [15], future research should be conducted to further extend and even develop more specific mechanisms of news consumption in relation to future pandemics. We hope that our research will encourage future researchers to conduct further research in the context of future pandemics, mortality salience, and mental health.

## 7 Conclusion

In conclusion, this study provides a nuanced understanding of how and when COVID‐19‐related knowledge mediates the relationship between COVID‐19‐related news consumption and young adults' mental health complaints. We specifically show that COVID‐19‐related news consumption is positively related to COVID‐19‐related knowledge, which eventually relates to mental health complaints, especially when COVID‐19‐triggered mortality salience is relatively high. We hope our study will spur future research into the future global pandemic and mental health.

## Author Contributions

**H. M. Saidur Rahaman:** conceptualization, formal analysis, writing – original draft, writing – review and editing, supervision. **Rozi Samad:** project administration, methodology, data curation, writing – original draft.

## Conflicts of Interest

The authors declare no conflicts of interest.

## Transparency Statement

The lead author, H. M. Saidur Rahaman, affirms that this manuscript is an honest, accurate, and transparent account of the study being reported; that no important aspects of the study have been omitted; and that any discrepancies from the study as planned (and, if relevant, registered) have been explained.

## Data Availability

Data can be made available based on reasonable requests from the corresponding author.
